# A Highly Sensitive and Selective Fluorescent Probe Using MPA-InP/ZnS QDs for Detection of Trace Amounts of Cu^2+^ in Water

**DOI:** 10.3390/foods10112777

**Published:** 2021-11-11

**Authors:** Zeyu Xu, Yizhong Wang, Jiaran Zhang, Ce Shi, Xinting Yang

**Affiliations:** 1 College of Electronic Information and Automation, Tianjin University of Science and Technology, Tianjin 300222, China; xzy19950811@163.com (Z.X.); yzwang@tust.edu.cn (Y.W.); 2 Information Technology Research Center, Beijing Academy of Agriculture and Forestry Sciences, Beijing 100097, China; shice001@163.com (C.S.); yangxt@nercita.org.cn (X.Y.); 3 National Engineering Research Center for Information Technology in Agriculture, Beijing Academy of Agricultural and Forestry Sciences, Beijing 100097, China; 4 National Engineering Laboratory for Agri-Product Quality Traceability, Beijing Academy of Agricultural and Forestry Sciences, Beijing 100097, China

**Keywords:** fluorescence, quantum dots, copper (II) ions, water

## Abstract

Detection of copper (II) ions (Cu^2+^) in water is important for preventing them from entering the human body to preserve human health. Here, a highly sensitive and selective fluorescence probe that uses mercaptopropionic acid (MPA)-capped InP/ZnS quantum dots (MPA-InP/ZnS QDs) was proposed for the detection of trace amounts of Cu^2+^ in water. The fluorescence of MPA-InP/ZnS QDs can be quenched significantly in the presence of Cu^2+^, and the fluorescence intensity shows excellent linearity when the concentration of Cu^2+^ varies from 0–1000 nM; this probe also exhibits an extremely low limit of detection of 0.22 nM. Furthermore, a possible fluorescence-quenching mechanism was proposed. The MPA-InP/ZnS QDs probes were further applied to the detection of trace Cu^2+^ in real water samples and drink samples, showing good feasibility.

## 1. Introduction

As one of the essential trace elements for humans, Cu^2+^ plays an important role in many physiological processes, such as hemoglobin regulation, bone formation, and cell metabolism [1]. However, high concentrations of Cu^2+^ can affect the function of the kidney and liver and may cause brain diseases, such as Alzheimer’s disease and Parkinson’s disease [2]. Even short-term exposure to high concentrations of Cu^2+^ can cause stomach and intestinal discomfort [3]. Cu^2+^ is taken up by humans through contaminated water and food and accumulates in the human body. To this end, the maximum residue levels of Cu^2+^ in water are clearly defined. The United States Environmental Protection Agency (USEPA) stipulated that the maximum allowable amounts of Cu^2+^ in drinking water and in industrial effluents are 20 μM [4] and 1.3 mg/L [5], respectively. Therefore, detecting Cu^2+^ in water is of great significance for preventing Cu^2+^ from entering the human body and ensuring human health.

There are many methods for detecting Cu^2+^, such as atomic absorption spectrometry [6], inductively coupled plasma optical emission spectrometry (ICP-OES) [7], inductively coupled plasma mass spectrometry (ICP-MS) [8], electrochemical methods [9], and fluorescence methods [10]. Atomic absorption spectrometry, ICP-OES, and ICP-MS are highly accurate for the detection of Cu^2+^; however, the disadvantages of expensive equipment, cumbersome operation, and time consumption [11,12] make them unable to detect mobile Cu^2+^ in real time. Electrochemical methods have the advantages of low cost, simple operation, and portability; however, the electrodes have to be maintained due to the polarization effect [13]. Fluorescence methods have attracted increasing attention in the field of Cu^2+^ detection in recent years due to their high sensitivity, anti-interference ability, and fast response. In particular, with the development of fluorescent nanomaterials, Cu^2+^ fluorescent strategies with simple and environmentally friendly designs have attracted increasing interest [14,15].

Quantum dots (QDs) are widely used in the detection of Cu^2+^ in water due to their advantages of simple preparation, stable optical performance, adjustable emission, and surface modification [16]. Sadeghi et al. [17] developed a fluorescent probe based on CdSe QDs capped with deep eutectic solvent (DES-CdSe QDs) to determine the levels of Cu^2+^ in various drinks based on aggregation-induced emission (AIE), but CdSe QDs are poisonous and environmentally unfriendly, which limits the application of the method. Carbon dots (CDs) are some of most popular QDs in the detection of Cu^2+^ [18]. The fluorescence of CDs can be quenched in the presence of Cu^2+^ based on the complexation reaction of Cu^2+^ with the oxygen-containing functional groups on the surface of the CDs. Although CDs have the advantages of simple synthesis, low price, and nontoxicity, some metal ions that could be present in the water, such as iron ions and mercury ions, can also quench the fluorescence of the CDs [19,20], which can interfere with the detection of Cu^2+^. Therefore, in real environmental samples, eliminating the influence of potential interfering substances to detect Cu^2+^, even in trace amounts and with highly sensitive methods, is one of the main challenges.

In this work, we synthesized 3-mercaptopropionic acid (MPA)-capped InP/ZnS QDs (MPA-capped InP/ZnS QDs) for the first time and developed a fluorescent probe based on MPA-InP/ZnS QDs for the detection of trace Cu^2+^ in water. With proper capping of MPA, the MPA-InP/ZnS QDs exhibit excellent monodispersity in aqueous media and are suitable for the highly selective detection of trace Cu^2+^. In the presence of Cu^2+^, the fluorescence of MPA-InP/ZnS QDs could be significantly quenched, the sensing performance was studied, and the possible mechanism was proposed. Furthermore, the probe was applied to the detection of Cu^2+^ in environmental water and drink samples. To the best of our knowledge, there are few reports on the use of MPA-InP/ZnS QDs to detect Cu^2+^ in water.

## 2. Materials and Methods

### 2.1. Materials and Instruments

Indium (III) iodide (InI_3_) (99.998%), zinc (II) chloride (ZnCl_2_) (≥98%), tris(diethylamino)-phosphine (C_12_H_30_N_3_P) (97%), 100 mesh selenium powder (99.99%), hydrochloric acid (HCI) (mass fraction, 36.46%), zinc stearate (C_36_H_70_O_4_Zn) (technical grade, 65%), trioctylphosphine (C_24_H_51_P) (>97%), octadecene (C_18_H_36_) (technical grade, 90%), chloroform (CHCI_3_) (≥99%), 3-mercaptopropionic acid (C_3_H_6_O_2_S) (MPA) (≥99%), tetramethylammonium hydroxide pentahydrate ((CH_3_)_4_N-OH·5H_2_O) (TMAH) (≥97%), sulfur powder, oleylamine (C_18_H_37_N) (mass fraction, 80–90%), citric acid (C₆H₈O₇) (≥99.5%), disodium hydrogen phosphate (Na_2_HPO_4_) (≥99%), and all of the soluble metal salts (NaCl, MgCl_2_, AlCl_3_, KCl, CaCl_2_, MnCl_2_, FeCl_3_, CoCl_2_, CuCl_2_, PbCl_2_, CdCl_2_, BaCl_2_, and AgNO_3_) were analytically pure and purchased from Sigma–Aldrich (St. Louis, MO, USA). All chemical reagents were directly used without further purification. The distilled water used in all experiments had a conductivity of 1.37 (μs/cm) and a resistivity of 0.73 (MΩ/cm).

Transmission electron microscopy (TEM) observations were carried out by using a transmission electron microscope (JEOL, JEM-2100, Akishima, Japan). Fourier-transform infrared spectra (FT-IR) were obtained by FT-IR spectroscopy (Tianjin Jiangdong Company, FT-IR-650, Tianjin, China). X-ray photoelectron spectroscopy (XPS) was recorded by using an XPS spectrometer (Japan Electronics, D/MAX, Tokyo, Japan). Additionally, the quantum yield (QY) was measured by using a fluorescence spectrometer (Horiba Jobin Yvon, Nanolog FL3-2 IHR, Pairs, France). The fluorescence spectra of the probes were recorded by using a fluorescence spectrometer (Edinburgh Instruments Ltd., Edinburgh FS5, Livingston, UK) at an excitation wavelength (λex) of 250 nm and an emission wavelength (λem) of 520 nm with an integration time of 0.1 s. Both the excitation and emission slits were set to a width of 5 nm. A time-resolved fluorescence spectrometer (Horiba Jobinyvon IBH Inc, Deltaflex 77-500K, Pairs, France) was used to measure the fluorescence lifetime of the probes with λex = 250 nm and λem = 520 nm. The ultraviolet-visible (UV-Vis) spectrum was scanned in the wavelength range of 190–1100 nm with a UV-Vis spectrophotometer (Hach Company, DR6000, Loveland, USA) with a step size of 5 nm. A refrigerated centrifuge (Sigma, 3k15, Landkreis Osterode, Germany) was used for the pretreatment of samples. In addition, the pH was adjusted by an automatic acid-base titration apparatus (Mettler Toledo, Titration Excellence T9, Zurich, Switzerland), and the sample was sonicated by an ultrasonic cleaner (Kunshan Shumei, KQ-250DE, Kunshan, China).

### 2.2. Synthesis of MPA-InP/ZnS QDs

The MPA-InP/ZnS QDs were synthesized according to a previously reported procedure [21]. The solvothermal method was used to proceed with the MPA-InP/ZnS QDs. First, 100 mg of InI_3_ (0.45 mM) and 300 mg of ZnCl_2_ (2.2 mM) were added to 5.0 mL (15 mM) of technical oleylamine, which is a coordinating solvent at 120 °C, and stirred and degassed to react for one hour; then, 0.45 mL (1.6 mM) of tris(diethylamino)-phosphine (phosphorus:indium ratio = 3.6:1) was injected into the above mixture at 180 °C under inert atmosphere for 20 min to synthesize the InP nanocrystals. Subsequently, 1 mL of saturated TOP-S (2.2 M) was slowly injected into the solution of InP nanocrystals, and the temperature was raised to 200 °C after 40 min; 4 mL of ODE with 1 g Zn(stearate)_2_ was injected into the solution, and the temperature was increased to 220 °C after 60 min; 0.7 mL saturated TOP-S (2.2 M) was slowly injected into the solution, and the temperature was increased to 240 °C, and 2 mL of ODE with 0.5 g Zn(stearate)_2_ was injected into the solution in turn, and temperature was increased to 260 °C after 30 min and 60 min, respectively. At the end, the solution reacted at 260 °C for 30 min to form trioctyl phosphine oxide (TOPO)-capped InP/ZnS QDs.

To improve the monodispersion and optical properties in aqueous media, the InP/ZnS QDs were capped by MPA. One hundred milligrams of the organic base TMAH were mixed well vigorous shaking with 50 µL MPA in 1 mL of chloroform and allowed to stand for 1 h for MPA deprotonation. Then, the bottom organic phase containing deprotonated MPA was transferred into a polypropylene tube. Then, 100 µL of the TOPO-capped InP/ZnS QDs (0.1 µM in chloroform) was added to the polypropylene tube and mixed at room temperature for 40 h for the ligand-exchange reaction between TOPO and MPA. Finally, the product was washed with chloroform and ethanol 3 times to obtain MPA-InP/ZnS QDs.

### 2.3. Detection of Cu^2+^ Based on MPA-InP/ZnS QDs

Next, 100 μL of different concentrations of Cu^2+^ (0 nM, 3 nM, 5 nM, 10 nM, 15 nM, 20 nM, 30 nM, 50 nM, 100 nM, 150 nM, 200 nM, 250 nM, 300 nM, 400 nM, 600 nM, and 1000 nM) were added to 2 mL of MPA-InP/ZnS QDs PBS solution (14 nM, pH = 8.0) for 12 min incubation at room temperature, and the fluorescence spectra were recorded (λex = 250 nm). The calibration curves of the fluorescence intensity at the emission peak of 520 nm vs. the concentrations of Cu^2+^ were plotted.

The stability and selectivity of MPA-InP/ZnS QDs were investigated. The fluorescence intensity was recorded at indoor environment every day for 7 days at room temperature; different metal ions, including Na^+^, Mg^2+^, Al^3+^, K^+^, Ca^2+^, Co^2+^, Mn^2+^, Fe^3+^, Ba^2+^, Cd^2+^, Pb^2+^, and Ag^+^ (500 nM for each), were added to the MPA-InP/ZnS QDs solution, and the fluorescence intensity of each solution was recorded.

The probes were used for the detection of Cu^2+^ in the environmental water samples and drinking samples. In the spiked and real samples detection, 100 μL of samples were added to 2 mL of MPA-InP/ZnS QDs PBS solution (14 nM, pH = 8.0) for 12 min incubation at room temperature, and the fluorescence spectra were recorded (λex = 250 nm).

Samples 1–5 were mineral water of different brands, including Sample 1 (Uni-President; Tainan, China), Sample 2 (Jingtian Food & Beverage Co., Ltd.; Shenzhen, China), Sample 3 (Evergrande Mineral Water Group Co., Ltd.; Guangzhou, China), Sample 4 (Voss Beverage Co., Ltd.; Voss, Norway), and Sample 5 (Danone Co., Ltd.; Pairs, France); the carbonated drinks included Sample 6 (Coca-Cola; Atlanta, GA, USA) and Sample 7 (Pepsi; New York, NY, USA), and the drinking water Sample 8 (Beijing Xiangshan Tianquan Beverage Co., Ltd.; Beijing, China), all of which were purchased from the local market in Haidian District of Beijing. The river water samples (Sample 9) were obtained from the Jingmi Diversion Canal in Beijing. Tap water (Sample 10) was obtained in Haidian District of Beijing. The river water samples were centrifuged at 5000 rpm for 3 min to obtain the supernatant, which was filtered through a 0.45-μm filter membrane. The carbonated drink samples were pretreated by ultrasonic oscillation for 5 min. The Cu^2+^ of the samples were detected by ICP-MS method as comparisons [22] and all experiments were performed in triplicate. Unless otherwise specified, all experiments in this article were performed at room temperature.

## 3. Results and Discussion

### 3.1. Characterization of MPA-InP/ZnS QDs

**Figure 1:**
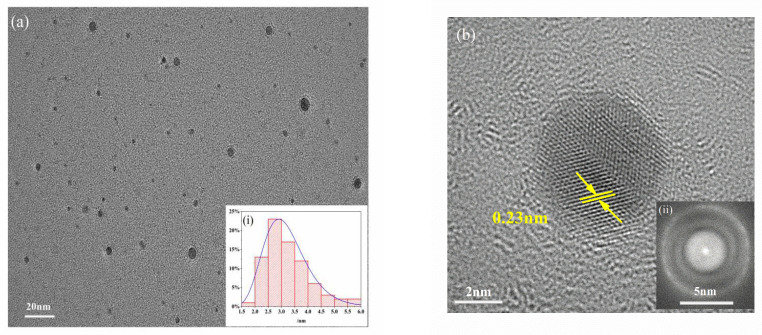
TEM (**a**) and HRTEM images (**b**) of MPA-InP/ZnS QDs (inset: particle size distribution (**i**) and CCD image of MPA-InP/ZnS QDs (**ii**)). TEM: transmission electron microscopy, HRTEM: high resolution transmission electron microscope, MPA-InP/ZnS QDs: mercaptopropionic acid capped InP/ZnS quantum dots, CCD: charge couple device.

As shown in Figure 1, the synthesized MPA-InP/ZnS QDs were spherical and well dispersed in water solution. The particle size of MPA-InP/ZnS QDs was distributed in a relatively narrow range of 1.87–5.86 nm, with an average size of 3.21 nm (inset in Figure 1a). The MPA-InP/ZnS QDs had an obvious lattice with a lattice spacing of 0.23 nm, as shown in Figure 1b, and the charge couple device (CCD) image revealed that InP/ZnS QDs have round shape and excellent light-emitting characteristics.

**Figure 2:**
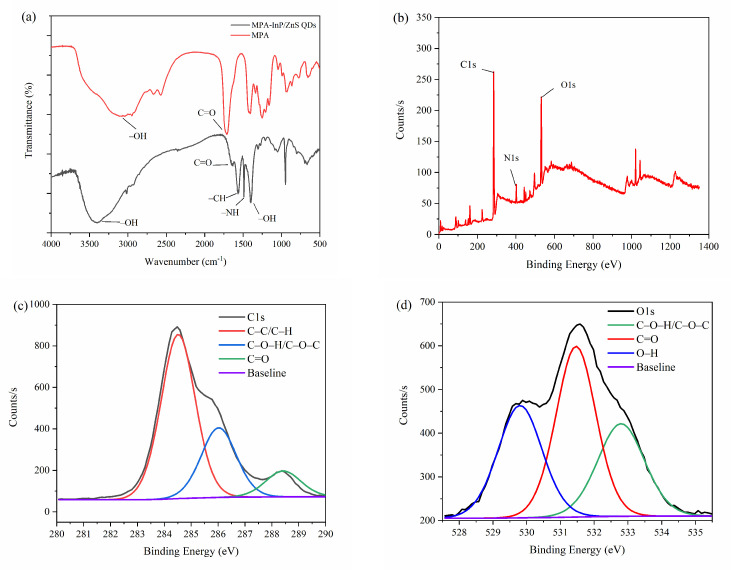
FT-IR spectrum (**a**), XPS patterns (**b**), C1s XPS spectrum (**c**), and O1s XPS spectrum (**d**) of MPA-InP/ZnS QDs. XPS: X-ray photoelectron spectroscopy, C1s XPS: X-ray photoelectron spectroscopy of 1s orbital electron peak of carbon atom, O1s XPS: X-ray photoelectron spectroscopy of 1s orbital electron peak of oxygen atom, MPA-InP/ZnS QDs: mercaptopropionic acid capped InP/ZnS quantum dots.

FT-IR spectra were obtained to investigate the functional groups on the surface of MPA-InP/ZnS QDs. As shown in Figure 2a, the FT-IR spectra of MPA (red curve) displays that the absorption bands at 3142 cm^−1^ and 1701 cm^−1^ are attributed to the stretching vibration of the O–H and the C=O of COOH, respectively. The FT-IR spectra of MPA-InP/ZnS QDs (black curve) shows the absorption band at 3396 cm^−1^ is attributed to the stretching vibration of the O–H band of COOH. The peaks at 1640, 1568, 1486, and 1396 cm^−1^ are caused by the stretching vibrations of the C=O, C–H, –NH, and –OH bonds, respectively. The presence of carboxyl hydroxyl and amine groups was demonstrated on the surface of the MPA-InP/ZnS QDs [23], which are beneficial to the homogenous and stable dispersion of MPA-InP/ZnS QDs in water [24] and indicating the InP/ZnS QDs are successfully capped by MPA. XPS spectra were recorded to investigate the surface elements of the MPA-InP/ZnS QDs. As shown in Figure 2b, the XPS spectrum of MPA-InP/ZnS QDs shows three peaks at 284.8, 402.4, and 532.0 eV, which correspond to the C1s, N1s, and O1s orbitals with relative atomic percentages of 73.96%, 5.01%, and 21.03%, respectively. The high-resolution C1s and O1s XPS spectra, as shown in Figure 2c,d, demonstrate the presence of C–C/C–H, C–O–H/C–O–N, O–H, and C=O, which agrees with the FT-IR spectra. The XPS spectra shows that the MPA-InP/ZnS QDs contain a large number of carboxyl groups, which further indicates that the InP/ZnS QDs are successfully capped by MPA.

**Figure 3:**
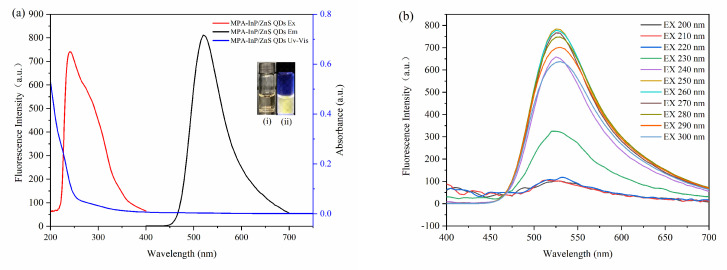
The absorption spectrum, excitation, and emission spectroscopy (**a**). (insert: photos of MPA-InP/ZnS QDs solutions under sunlight (**i**) and UV light (365 nm)) (**ii**) Emission spectroscopy with different excitation of MPA-InP/ZnS QDs (**b**). MPA-InP/ZnS QDs: mercaptopropionic acid capped InP/ZnS quantum dots.

The fluorescence excitation and emission spectra of the MPA-InP/ZnS QDs were obtained to investigate the fluorescence properties. As shown in Figure 3a, the MPA-InP/ZnS QDs show an emission wavelength at 520 nm with an excitation wavelength of 250 nm. The MPA-InP/ZnS QDs are pale yellow in daylight and exhibit strong yellow color under UV light (365 nm), as shown in the insert of Figure 3a. An obvious emission peak located at approximately 520 nm with excitation wavelength scanning from 200 nm to 300 nm can be observed in Figure 3b, indicating that the emission is independent of the excitation wavelength. The quantum yield of purified MPA-InP/ZnS QDs was measured by a fluorescence spectrometer with an integrating sphere to be 12.05%.

### 3.2. Detection of Cu^2+^ by Utilizing the MPA-InP/ZnS QDs

**Figure 4:**
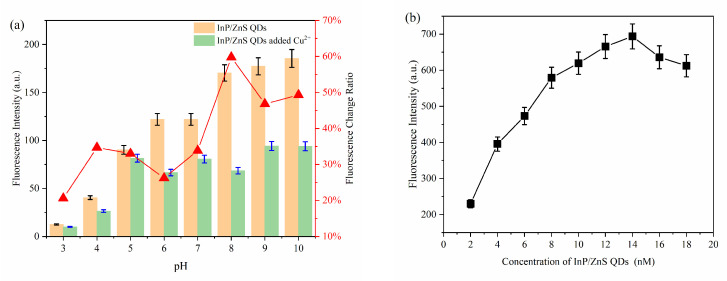

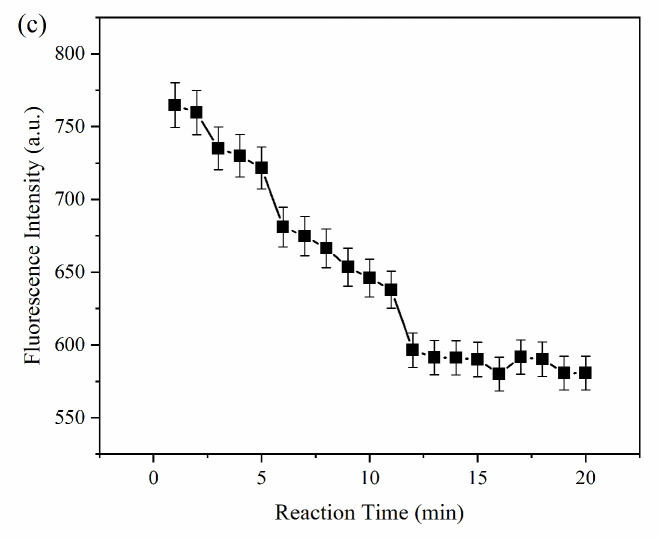
The fluorescence intensity of MPA-InP/ZnS QDs without and with Cu^2+^ in different pH and the fluorescence-quenching rate of MPA-InP/ZnS QDs after adding Cu^2+^ (**a**), the fluorescence intensity of MPA-InP/ZnS QDs in different concentrations (**b**), and the changes of fluorescence intensity of MPA-InP/ZnS QDs with added copper over time (**c**). MPA-InP/ZnS QDs: mercaptopropionic acid capped InP/ZnS quantum dots.

The influence of pH, the concentration of MPA-InP/ZnS QDs, and the reaction time were investigated to optimize the conditions of reaction conditions for the detection of Cu^2+^. As shown in Figure 4a, the ratio of the fluorescence quenching of the MPA-InP/ZnS QDs with Cu^2+^ (50 nM, 100 μL) added in PBS buffer at different pH values (2–10) was recorded, and the fluorescence was quenched most obviously at pH = 8.0. Thus, the value of 8.0 was used as the optimum pH. Figure 4b shows that the fluorescence intensity of MPA-InP/ZnS QDs at a concentration of 14 nM was the strongest, which means that the quenching degree may be the greatest in the presence of same concentration of Cu^2+^. In order to investigate this issue further, the fluorescence intensity of MPA-InP/ZnS QDs in the concentration of 6 nM, 10 nM, 14 nM, and 18 nM in the presence of different concentrations of Cu^2+^ were measured. As shown in Figure S1 of Supplementary Materials, in the concentrations of Cu^2+^ of 0–1000 nmol/L, the MPA-InP/ZnS QDs solution with a concentration of 14 nM has the greatest degree of fluorescence quenching. The results indicated that 14 nM is the best concentration. Furthermore, the time-based fluorescence behavior of the MPA-InP/ZnS QDs with added Cu^2+^ was studied, and it is shown in Figure 4c that the fluorescence was stable after 12 min. Therefore, we chose 12 min as the incubation time for the complete reaction of MPA-InP/ZnS QDs and Cu^2+^. All the following experiments were performed under the optimum conditions.

**Figure 5:**
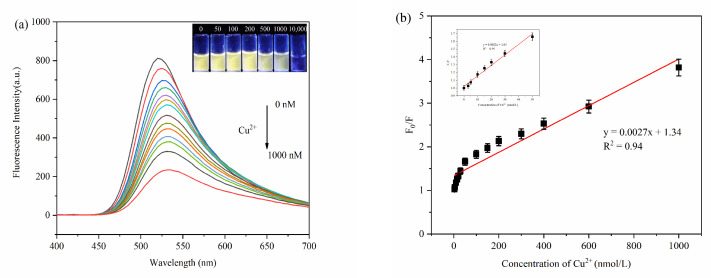
The fluorescence spectra of MPA-InP/ZnS QDs with different added concentrations of Cu^2+^ (0 nM, 3 nM, 5 nM, 10 nM, 15 nM, 20 nM, 30 nM, 50 nM, 100 nM, 150 nM, 200 nM, 250 nM, 300 nM, 400 nM, 600 nM, and 1000 nM) (**a**), (insert: photos of MPA-InP/ZnS QDs solutions with different concentrations of Cu^2+^ under 365 nm UV light) and the relationship between the F_0_/F and the concentrations of Cu^2+^ (**b**), (insert: the relationship between the F_0_/F and low concentrations of Cu^2+^ (0–50 nM)). MPA-InP/ZnS QDs: mercaptopropionic acid capped InP/ZnS quantum dots.

The fluorescence intensity of MPA-InP/ZnS QDs solutions with different concentrations of Cu^2+^ was measured at λex = 250 nm to explore the sensitivity in terms of detecting Cu^2+^ in water. Figure 5a shows a remarkable decrease in fluorescence intensity at 520 nm with increasing concentrations of Cu^2+^. The insets of Figure 5a shows photos of MPA-InP/ZnS QD solutions with different concentrations of Cu^2+^ irradiated by a UV lamp (365 nm). The color of the MPA-InP/ZnS QDs solution under UV light gradually changed from extremely bright yellow to pale yellow until colorless with increasing Cu^2+^ concentration. The Figure 5b shows a good linear relationship (R^2^ = 0.94) between the F_0_/F (F_0_ is the initial fluorescence intensity of MPA-InP/ZnS QDs, and F is the fluorescence intensity of MPA-InP/ZnS QDs after adding Cu^2+^) and concentration of Cu^2+^ in the range of 0–1000 nM. Moreover, the insert of Figure 5b shows that the fluorescence intensity of the MPA-InP/ZnS QDs at low concentrations of Cu^2+^ (0–50 nM) is quenched more rapidly, which means that the Cu^2+^ detection is extremely sensitive at low concentrations. In the concentration range of 0–50 nM, the calibration curve has better linearity (R^2^ = 0.99), and the limit of detection (LOD) of the prepared MPA-InP/ZnS QDs calculated by the Formula 3σ/x was 0.22 nM [18].

**Figure 6:**
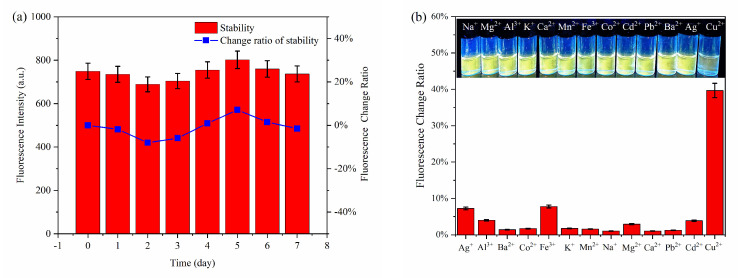
The stability (**a**) and the selectivity (**b**) of the fluorescent probe.

The stability and selectivity are important indicators for evaluating the practicability and feasibility of probes. Figure 6a shows that the changes in the fluorescence intensity of the MPA-InP/ZnS QDs solution at indoor environment are less than 8% within seven days, indicating the perfect stability of the MPA-InP/ZnS QDs probe. Furthermore, the influence of the potentially competing metal ions Na^+^, Mg^2+^, Al^3+^, K^+^, Ca^2+^, Co^2+^, Mn^2+^, Fe^3+^, Ba^2+^, Cd^2+^, Pb^2+^, and Ag^+^ (500 nM for each) was studied. As shown in Figure 6b, compared with Cu^2+^, only Ag^+^ and Fe^3+^ can quench the fluorescence of InP/ZnS QDs. The fluorescence-quenching degree of InP/ZnS QDs are 7.25% and 7.77% in the concentration of 500 nM of Ag^+^ and Fe^3+^, respectively. While in the presence of 50 nM Cu^2+^, the fluorescence-quenching degree of InP/ZnS QDs is 39.2%, indicating the fluorescence quenching of MPA-InP/ZnS QDs by other metal ions is almost negligible. The ion selectivity is dependent on the intrinsic affinity between the analyte and the surface ligands [25], and the MPA ligand has a high affinity constant with Cu^2+^ [26], which may be the reason of MPA-InP/ZnS QDs has higher selectivity to Cu^2+^ than other metal ions. These experimental results show the excellent sensitivity, high selectivity, and good anti-influence of the MPA-InP/ZnS QDs probe for the detection of trace Cu^2+^ in water.

### 3.3. Fluorescence-Quenching Mechanism of MPA-InP/ZnS QDs

**Figure 7:**
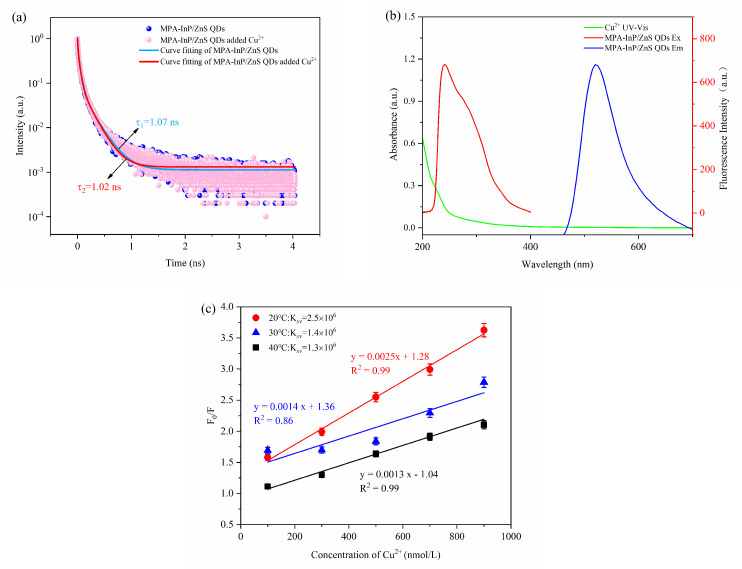
The fluorescence lifetime decay curves of MPA-InP/ZnS QDs without and with Cu^2+^ (**a**); the absorption spectrum of Cu^2+^, excitation, and emission spectra of MPA-InP/ZnS QDs (**b**); the calibration curves between F_0_/F and the concentrations of Cu^2+^ under different temperature (20 °C, 30 °C, and 40 °C) (**c**). MPA-InP/ZnS QDs: mercaptopropionic acid capped InP/ZnS quantum dots.

The fluorescence-quenching mechanism of quantum dots is complicated, generally including inner filter effect (IFE), fluorescence resonance energy transfer (FRET), photoinduced electron transfer (PET), static quenching effect (SQE), and dynamic quenching. The FRET, PET, and dynamic quenching can cause the fluorescence lifetime of the fluorophore to decay after adding a quencher, while IFE and SQE cannot [27]. As shown in Figure 7a, the fluorescence decay lifetimes of the MPA-InP/ZnS QDs without and with Cu^2+^ were 1.07 ns and 1.02 ns, respectively; these values were almost unchanged, indicating that FRET, PET, and dynamic quenching do not occur between MPA-InP/ZnS QDs and Cu^2+^. Moreover, IFE can be confirmed by UV-Vis absorption spectrum because IFE requires a certain degree of overlap between the excitation or emission band of the fluorophore and the absorption band of the UV-Vis spectrum of the quencher [28]. As shown in Figure 7b, the absorption band of Cu^2+^ in the range of 200 nm to 270 nm with a peak at 205 nm was observed, and the absorption band of Cu^2+^ overlaps with the excitation peak of the MPA-InP/ZnS QDs at 250 nm, indicating that the IFE may exist in the quenching mechanism. To further confirm the quenching mechanism, a typical Stern–Volmer diagram was constructed:

(1) $$F_{0}/F=1+K_{SV}\left[Q\right]=1+K_{q}\tau_{0}\left[Q\right]$$

where *F*_0_ is the fluorescence intensity of MPA-InP/ZnS QDs, *F* is the fluorescence intensity observed after adding Cu^2+^, *K_SV_* is the quenching Stern–Volmer constant, and [*Q*] is the concentration of the Cu^2+^, *K_q_* is the dynamic quenching Stern–Volmer constant, and $\tau_{0}$ is the lifetime of the InP/ZnS QDs. The static quenching can be judged by *K_SV_* in different temperatures. The value of *K_SV_* decreases with the increase of temperature in the SQE process [29]. Obviously, as shown in Figure 7c, with the increasing temperature from 20 °C to 40 °C, the slope of the curves that are *K_SV_* decreases, which confirms the existence of SQE. Moreover, the maximum *K_q_* of collision quenching is 2 × 10^10^ L mol^−1^ s^−1^ [30]. In addition, the *K_q_* of 20 °C, 30 °C, and 40 °C in this paper are 2.34 × 10^15^, 1.31 × 10^15^, and 1.21 × 10^15^, respectively, which are much larger. The results further confirm the existence of SQE.

### 3.4. Detection of Cu^2+^ in Real Samples

The applicability and accuracy of MPA-InP/ZnS QDs for the detection of Cu^2+^ in environmental water samples and drinking samples were investigated. As shown in Table 1, spiked detections were carried out in pure water of Watsons; the recovery was 93.64–120.91%, and the relative standard deviation (RSD) (*n* = 3) was below 1.02%. The results of the detection of Cu^2+^ in different water samples using our prepared MPA-InP/ZnS QDs probes and ICP-MS are listed in Table 2 and Figure S2. The results of the two methods demonstrate that the MPA-InP/ZnS QDs probes are highly accurate and have the ability to detect trace amounts of Cu^2+^ in real water samples.

The performance of MPA-InP/ZnS QDs probes was also compared with several previously reported studies on the detection of Cu^2+^ in water. As shown in Table 3 and Figure S3, the detection range of fluorescent probes for Cu^2+^ can reach hundreds of micromoles; however, the LOD is difficult to reach for the nanomolar level. As the comparison, the detection range of our probe is 0–1000 nM with the LOD of 0.22 nM, which exhibited superior sensing performance, especially in the detection of trace Cu^2+^. Our work shows promising prospects in the highly sensitive detection of trace Cu^2+^ in real water.

## 4. Conclusions

In summary, MPA-capped MPA-InP/ZnS QDs were synthesized by a solvothermal method; they exhibited bright yellow fluorescence and excellent monodispersity in aqueous solution. A highly sensitive and highly selective fluorescent probe for the detection of trace Cu^2+^ was developed using the synthesized MPA-InP/ZnS QDs. The fluorescence intensity of the MPA-InP/ZnS QDs can be quenched significantly in the presence of Cu^2+^ due to the SQE, which showed an excellent linear relationship with Cu^2+^ in the concentration range of 0–1000 nM, with a detection limit of 0.22 nM. Furthermore, the probe was applied to the detection of Cu^2+^ in environmental water and drink samples, indicating that MPA-InP/ZnS QDs could be used as a fluorescence probe in the application of highly sensitive and rapid detection of trace Cu^2+^ in real water.

## Figures and Tables

**Table 1:** Determination results of copper ions (Cu^2+^) in pure water of Watsons samples (*n* = 3).

Spiked/nM	Found/nM	Recovery (%)	RSD (%)
5.00	4.97	99.33	0.58
10.00	12.09	120.91	0.54
20.00	18.73	93.64	1.02

**Table 2:** Detection of Cu^2+^ using this method and ICP-MS (*n* = 3).

Samples	This Method		ICP-MS	
	Found/nM	RSD (%)	Found/nM	RSD (%)
Sample 1	5.16	5.24	5.31	0.54
Sample 2	4.29	2.42	3.75	1.04
Sample 3	14.03	1.96	12.50	0.67
Sample 4	7.97	1.09	6.56	0.47
Sample 5	13.59	1.79	14.69	0.64
Sample 6	4.68	5.66	3.38	1.23
Sample 7	5.07	7.25	3.69	0.78
Sample 8	3.81	2.96	3.75	0.97
Sample 9	17.47	4.47	15.09	1.06
Sample 10	31.06	5.13	33.13	0.54

**Table 3:** Comparison of the performance of fluorescent MPA-InP/ZnS QDs probes for the detection of Cu^2+^.

Materials	Principle	Detection Range	LOD	Reaction Time	Applications	Ref.
Mercaptoacetic acid-CdTe QDs	Dynamic quenching	40–600 nM	35.0 nM	5 min	Urine	[31]
Polyamine@C-dots	Static quenching effect	0.07–60 μM	0.02 μM	15 min	Conduit water, tap water, and mineral water	[18]
Coumarin	Static quenching effect	0–50 μM	0.27 μM	10 min	Tap water	[32]
CdTe QDs-polyethyleneimine/ polyvinyl alcohol electrospun	Dynamic quenching	0.08–800 μM	11.1 nM	50 min	Lake water	[26]
Deep eutectic solvent-CdSe QDs	Aggregation-induced emission	10–600 nM	5.3 nM	1 min	Tap water, mineral water, pineapple fruit juice, milk, and cola	[17]
MPA-InP/ZnS QDs	Static quenching effect	0–1000 nM	0.22 nM	12 min	River water, tap water, purified water, mineral water, drinking water, and beverages	This work

MPA-InP/ZnS QDs: mercaptopropionic acid capped InP/ZnS quantum dots.

## Data Availability

Not applicable.

## Supplementary Materials

The following are available online at https://www.mdpi.com/article/10.3390/foods10112777/s1, Figure S1: The response curves of InP/ZnS QDs solutions with concentrations of 6 nM, 10 nM, 14 nM and 18 nM to different concentrations of Cu^2+^, Figure S2: The data comparison image of the detection results of the two methods, Figure S3: The results comparison image of this work and literature.
