# Supplementary material for: A Highly Sensitive and Selective Fluorescent Probe Using MPA-InP/ZnS QDs for Detection of Trace Amounts of Cu2+ in Water
Source: Foods. 2021 Nov 11;10(11):2777. doi: 10.3390/foods10112777 (PMC8617727; doi:10.3390/foods10112777)
Supplement: Supplementary file 1 [file foods-10-02777-s001.zip › foods-1440229-supplementary.pdf]

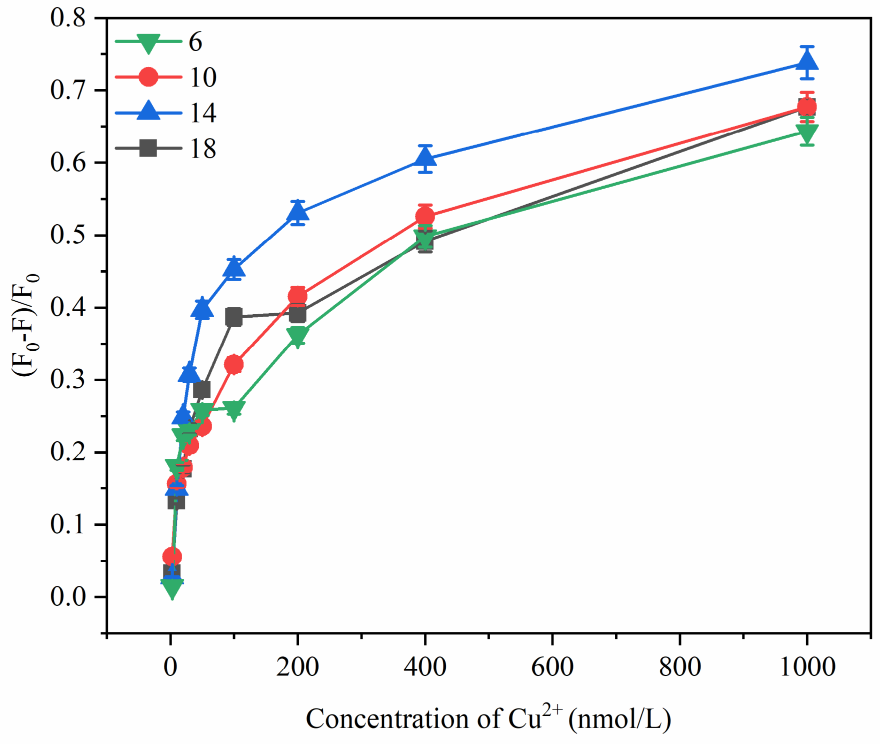

Figure S1. The response curves of InP/ZnS QDs solutions with concentrations of 6 nM, 10 nM, 14 nM and 18 nM to different concentrations of Cu^{2+}.

It can be seen from Fig. 4b that when the concentration of MPA-InP/ZnS QDs was 14nM, the fluorescence intensity is the strongest, which means that the quenching degree may be the greatest in the presence of same concentration of Cu^{2+}. In order to investigate this issue further, the fluorescence intensity of MPA-InP/ZnS QDs in the concentration of 6 nM, 10 nM, 14 nM, and 18 nM in the presence of different concentrations of Cu^{2+} were measured. As shown in Fig S1 of supplementary information, in the concentrations of Cu^{2+} of 0-1000 nmol/L, the MPA-InP/ZnS QDs solution with a concentration of 14nM has the greatest degree of fluorescence quenching. The results indicated that 14nM is the best concentration.

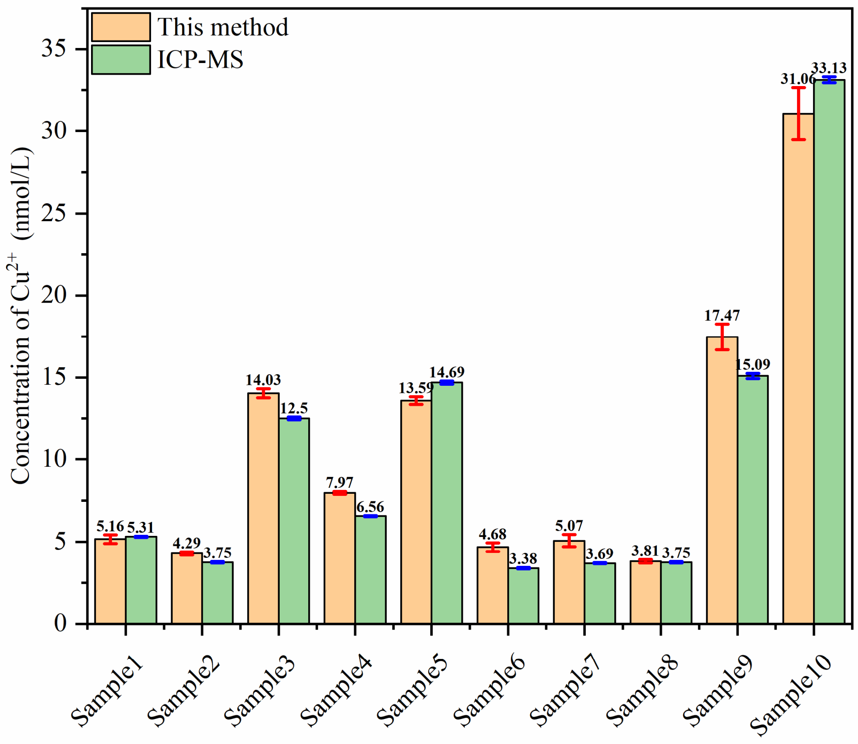

Figure S2. The data comparison image of the detection results of the two methods.

In the Fig. S2, all the samples used in MPA-InP/ZnS QDs probes and ICP-MS method were from the same batch and the same pretreatment methods. ICP-MS is a commonly used method for analyzing the content of metal ions, especially for the detection of trace amount of metal ions with high accuracy. In this paper, the detection results for the same sample by using the MPA-InP/ZnS QDs probe and the ICP-MS method are compared, aiming to show that the MPA-InP/ZnS QDs probe has good practicability and accuracy in the detection of trace amount of copper ions.

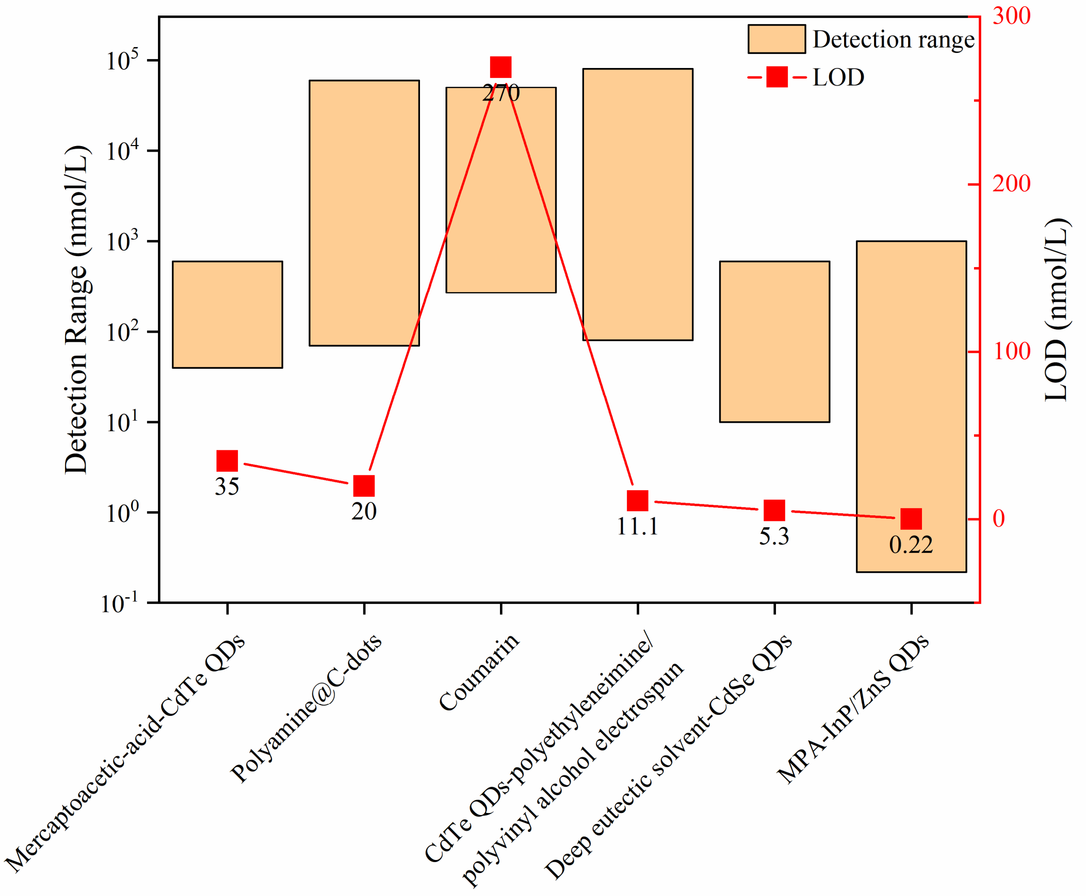

Figure S3. The results comparison image of this work and literature.

The results of this work are compared with the literature in Fig. S3. As shown in Table 3 and Fig. S3, the probe of CdTe QDs-polyethyleneimine/polyvinyl alcohol electrospun [26] has linearly response to Cu^{2+} in the range of 0.08–800 μM. The response time of the probe is 50 min,which limits its application in the field of rapid detection. The probe of coumarin [32] has linearly response to Cu^{2+} in the range of 0-50 μM. However, the LOD of the probe is 0.27 μM, which indicates the probe cannot be used in the detection of trace Cu^{2+}. The probe of deep eutectic solvent-CdSe QDs [17] has linearly response to Cu^{2+} in the range of 10-600 nM and the response time is 1 min. The probe detects Cu^{2+} by the mechanism of fluorescence recovery, which indicates the probe can avoid interference from other ions. However, CdSe QDs are poisonous and environmentally unfriendly, which cause harm to operators. Above all, the LOD of MPA-InP/ZnS

QDs for Cu^{2+} is as low as 0.22 nM in this work, far lower than that in other literature. It exhibits superior sensing performance, especially in the detection of trace Cu^{2+}.
